# Targeting the Monocyte–Macrophage Lineage in Solid Organ Transplantation

**DOI:** 10.3389/fimmu.2017.00153

**Published:** 2017-02-16

**Authors:** Thierry P. P. van den Bosch, Nynke M. Kannegieter, Dennis A. Hesselink, Carla C. Baan, Ajda T. Rowshani

**Affiliations:** ^1^Department of Internal Medicine, Section of Nephrology and Transplantation, Erasmus MC, University Medical Center Rotterdam, Rotterdam, Netherlands

**Keywords:** monocyte, macrophage, transplantation, immunosuppressive drug, signaling pathways

## Abstract

There is an unmet clinical need for immunotherapeutic strategies that specifically target the active immune cells participating in the process of rejection after solid organ transplantation. The monocyte–macrophage cell lineage is increasingly recognized as a major player in acute and chronic allograft immunopathology. The dominant presence of cells of this lineage in rejecting allograft tissue is associated with worse graft function and survival. Monocytes and macrophages contribute to alloimmunity *via* diverse pathways: antigen processing and presentation, costimulation, pro-inflammatory cytokine production, and tissue repair. Cross talk with other recipient immune competent cells and donor endothelial cells leads to amplification of inflammation and a cytolytic response in the graft. Surprisingly, little is known about therapeutic manipulation of the function of cells of the monocyte–macrophage lineage in transplantation by immunosuppressive agents. Although not primarily designed to target monocyte–macrophage lineage cells, multiple categories of currently prescribed immunosuppressive drugs, such as mycophenolate mofetil, mammalian target of rapamycin inhibitors, and calcineurin inhibitors, do have limited inhibitory effects. These effects include diminishing the degree of cytokine production, thereby blocking costimulation and inhibiting the migration of monocytes to the site of rejection. Outside the field of transplantation, some clinical studies have shown that the monoclonal antibodies canakinumab, tocilizumab, and infliximab are effective in inhibiting monocyte functions. Indirect effects have also been shown for simvastatin, a lipid lowering drug, and bromodomain and extra-terminal motif inhibitors that reduce the cytokine production by monocytes–macrophages in patients with diabetes mellitus and rheumatoid arthritis. To date, detailed knowledge concerning the origin, the developmental requirements, and functions of diverse specialized monocyte–macrophage subsets justifies research for therapeutic manipulation. Here, we will discuss the effects of currently prescribed immunosuppressive drugs on monocyte/macrophage features and the future challenges.

## Introduction

Solid organ transplantation (SOT) is the preferred method to treat organ failure. Over the past decades, transplantation has become the preferred approach to treat solid organ failure. Striking improvement in short-term allograft survival, in particular of kidney allograft, has been achieved, while long-term survival has lagged behind (1). Intriguingly, this improvement is seen mainly in recipients who have never experienced a rejection episode, thereby emphasizing the recipient’s alloimmunity, in particular chronic antibody-mediated rejection (cABMR) as a major determinant of overall transplant outcome (2, 3). At present, there is an unmet clinical need to apply immunotherapeutic strategies to specifically target the active immune cells crucially participating in the process of rejection after SOT.

However, treatment with immunosuppressive drugs has exchanged the morbidity and mortality of organ failure for the risks of infection, cancer, and increased mortality from cardiovascular disease. Although acute and chronic rejection, regardless of the type and the time of occurrence, are still major contributors leading to graft failure (1, 4, 5), cABMR is the main concern for the long-term graft survival. cABMR arises, at least in part, because immunosuppressive strategies do not completely inhibit rejection-related alloimmune responses specifically, thereby resulting in slow progressive deterioration of graft function.

The monocyte–macrophage cell lineage is increasingly recognized as a major player in acute and chronic allograft immunopathology (6, 7). The clinically used immunosuppressive drugs are not specifically directed against monocyte–macrophage lineage cells but still have some inhibitory effects. These cells contribute to alloimmunity *via* diverse pathways, antigen processing and antigen presentation, costimulation, pro-inflammatory cytokine production, and tissue repair. Cross talk with other recipient immune competent cells and donor endothelial cells underlies amplification of inflammation at the graft site (8–10). Interestingly, acute antibody-mediated rejection (ABMR) and cABMR are characterized among others by accumulation of monocyte–macrophage cells. Kidney graft-infiltrating macrophages have been described to be a predictor of death-censored graft failure (11–21). Macrophages are present in both acute ABMR and acute cellular rejection (ACR) of solid organ transplants (21, 22). In rejecting cardiac tissue, interstitial and intraluminal macrophage density correlates with effector alloantibodies and clinical ABMR (22). Even more, histopathological stainings for macrophages have been found to be positive prior to the onset of graft dysfunction indicating that macrophages can serve as potential diagnostic markers for transplant rejection (13). Intravascular macrophages in the capillaries of endomyocardial tissue are shown to be a distinguishing feature of ABMR and are considered as one of the important histopathological diagnostic criteria in cardiac transplantation (22, 23).

A recent study showed that the severity of macrophage infiltration during ACR with arthritis is associated with impaired kidney function as measured by creatinine values up to 36 months post-transplantation (21). Importantly, Oberbarnscheidt et al. showed that monocyte recognition of allogeneic non-self persists over time, long after acute surgical inflammation has been subsided, indicating the important role of monocytes in the principle of long-term graft failure (24). Recently, the presence of smooth muscle-like precursor cells within the non-classical monocyte subset has been described in kidney transplant patients. Characterization of non-classical monocytes in peripheral blood of kidney transplant patients undergoing chronic transplant dysfunction showed lower numbers compared to patients without chronic transplant dysfunction. Within the total living cell percentages of CD14^+^ monocytes, there was no change observed, suggesting a shift within different subsets. Non-classical monocytes being reduced in transplant recipients with chronic transplant dysfunction may indicate a vital role in interstitial and vascular remodeling (25).

In stable kidney transplant recipients, a skewed balance toward pro-inflammatory CD16^+^ monocytes was shown at the time of kidney transplantation and during the first 6 months post-transplant. These monocytes were able to produce IFNγ, which acts as an important bridge between innate and adaptive immunity (26, 27).

**Figure 1:**
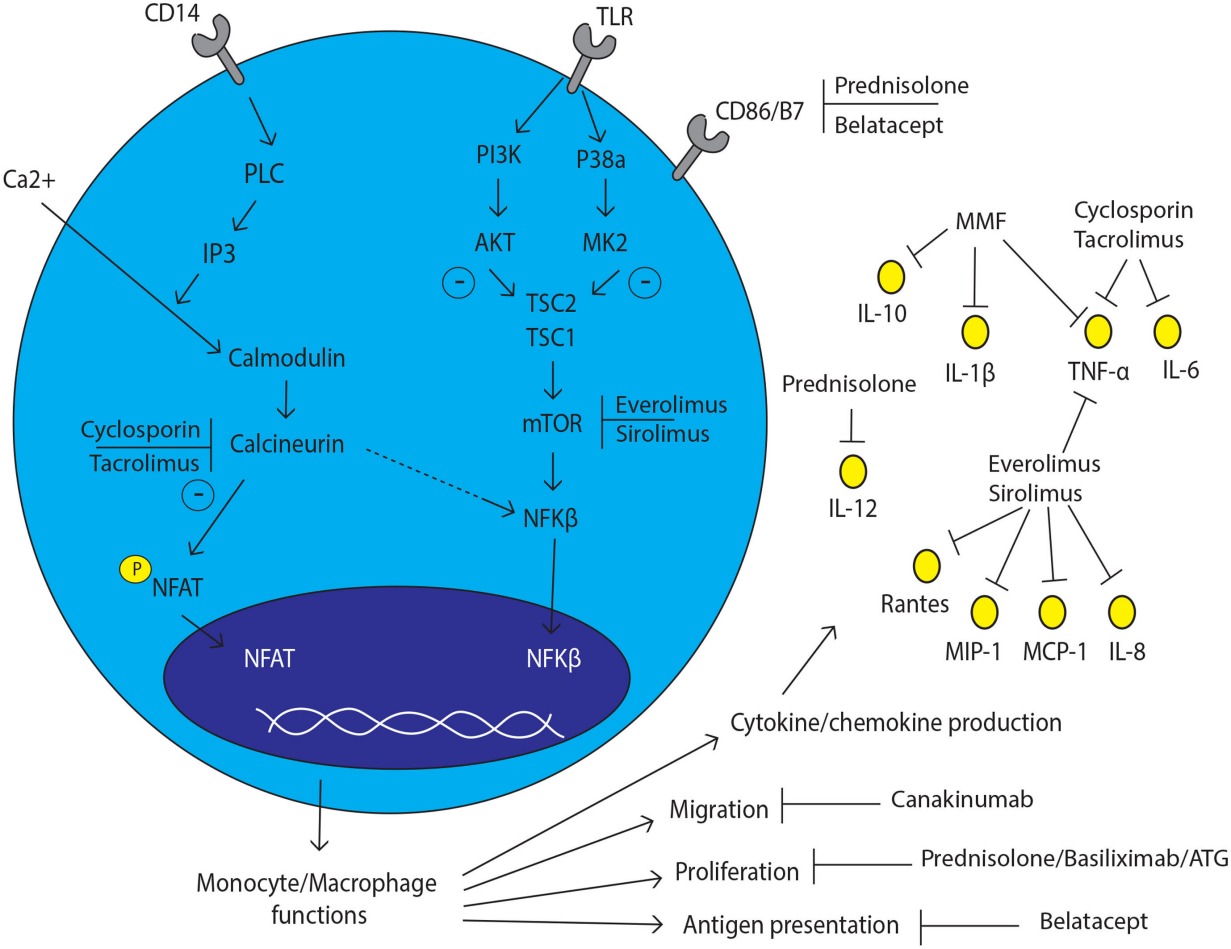
**Monocyte and macrophage lineage cell and the effect of immunosuppressive drugs**. The effect of currently prescribed immunosuppressive drugs with several inhibition spots on and in monocyte/macrophage lineage cells.

In summary, the currently available knowledge concerning the immunobiology of specialized monocyte–macrophage subsets, their pathogenic role in rejection, and the still unmet clinical need to specifically prevent alloimmunity justify research on strategies for monocyte–macrophage-directed therapeutics. In this review, we aim to discuss the relevant knowledge on monocyte–macrophage immunobiology briefly. To elaborate on the effects of currently available immunosuppressive drugs in relation to monocyte/macrophage lineage cells mainly focused within, but also outside of the SOT field (Table 1 and Figure 1), and eventually touch upon the future challenges and developments.

**Table 1:** **Immunosuppressive drugs and the monocyte/macrophage lineage**.

Drug type	Effects on monocytes/macrophages	Key reference
Basiliximab and ATG	Basiliximab targets the CD25 molecule (the IL-2 receptor) on activated T cells ATG binds to multiple T-cell-specific antigens and causes cell death *via* complement-mediated cytotoxicity Reduced number of monocytes *in vivo* Upregulation of the anti-inflammatory M2 macrophage subset CD14^+^CD163^+^ *in vivo*	Sekerkova et al. (28)
Alemtuzumab	Targets CD52 on B cells, T cells, NK cells, dendritic cells, and monocytes Less effective in depleting monocytes than depleting T cells Leads to a relative high expression of costimulatory molecules, such as IL-6 and NF-κB	Hale et al. (29), Kirk et al. (30), Fabian et al. (31), and Rao et al. (32)
Calcineurin inhibitors (tacrolimus and cyclosporine)	No inhibitory effect on p38MAPK phosphorylation, but reduce cytokine production *via* ERK phosphorylation Downregulate production of IL-6 and TNF-α after toll-like receptor stimulation *in vitro* Impaired phagocytosis function and promotion of infection (CsA)	Escolano et al. (33), Howell et al. (34), and Tourneur et al. (35)
Mycophenolate mofetil	Diminished the production of IL-1β, IL-10, and TNF-α and decreased expression of TNF-receptor 1 on monocytes Reduced monocyte migration through lower expression of adhesion molecules	Allison and Eugui (36) and Weimer et al. (37)
Glucocorticoids	Lower CD14^+^CD16^++^ monocyte counts Lower expression of B7 molecules leading to disturbed costimulation Induction of anti-inflammatory response *via* increased IL-10 production Impaired phagocytosis function	Rogacev et al. (38), Girndt et al. (39), Hodge et al. (40), Blotta et al. (41), and Rinehart et al. (42)
Mammalian target of rapamycin inhibitors	Decreased chemokine and cytokine production Combination therapy with steroids increased pro-inflammatory cytokine production	Lin et al. (43), Oliveira et al. (44), and Weichhart et al. (45)
Belatacept/abatacept	Block CD80/86 molecules on antigen-presenting cells and inhibit costimulatory function Lower migration and adhesion capacity Decreased expression of the pro-inflammatory cytokines such as IL-12 and TNF-α	Latek et al. (46), Bonelli et al. (47), and Wenink et al. (48)
Experimental drugs	Canakinumab inhibits IL-1β production by monocytes Sinomenine is associated with less monocyte migration, differentiation, and maturation 15-Deoxyspergualin decreases monocyte proliferation, TNF-α production, phagocytosis, and antigen presentation Simvastatin and salsalate are associated with less monocyte activation and inhibition of IL-6 and IL-8 production in diabetes patients Tocilizumab inhibits IL-6 production by monocytes BET inhibitors are involved in epigenetic control of monocytes thereby preventing inflammation Fish oils are associated with lower numbers of macrophages in obesitas patients and a reduced secretion of TNF-α *in vitro*	Hoffmann et al. (49), Ou et al. (50), Wang and Li (51), Perenyei et al. (52), Donath and Shoelson (53), McCarty (54), Tono et al. (55), Chan et al. (56), Spencer et al. (57), Zhao et al. (58), and Jialal et al. (59)

*ATG, antithymocyte globulin; IL, interleukin; NF-κB, nuclear factor kappa-light-chain enhancer of activated B cells; MAPK, mitogen-activated protein kinase; ERK, extracellular signal-regulated kinase; CsA, cyclosporin A; TNF, tumor necrosis factor; BET, bromodomain and extra-terminal motif*.

## Monocyte Immunobiology

**Figure 2:**
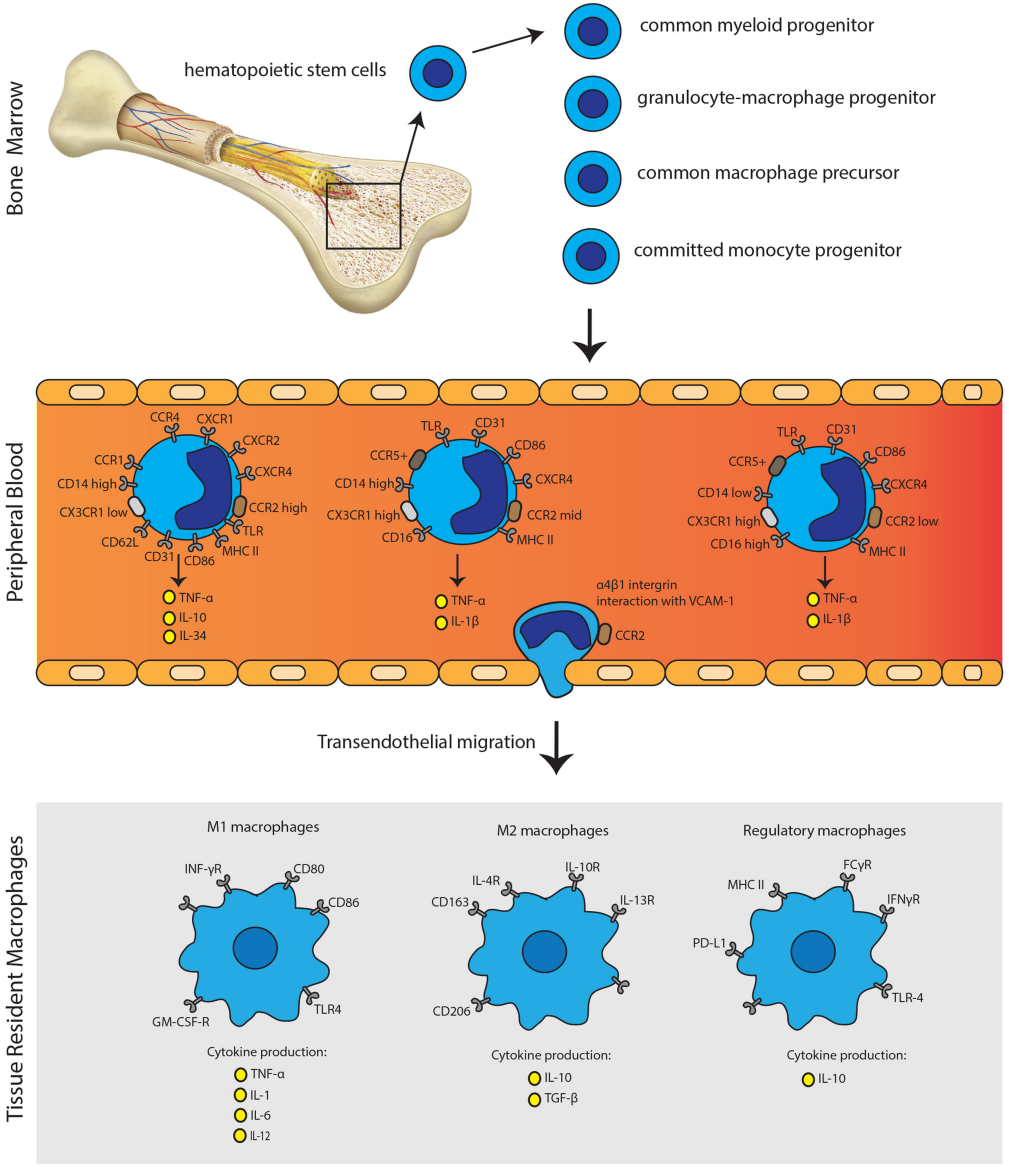
**Monocyte immunobiology**. Monocytes arise from myeloid precursor cells in primary lymphoid organs, including liver and bone marrow. In the peripheral blood, monocytes can be subdivided into three distinct subsets according to their CD14 and CD16 expression profile. Monocytes can undergo transendothelial migration through α4β1 integrin interaction with VCAM-1. Activation of monocytes is followed by the polarization of macrophages to acquire pro-inflammatory phenotype (M1), anti-inflammatory phenotype (M2) or the regulatory phenotype (Mreg). The secretion of distinct pro-inflammatory or anti-inflammatory cytokines, next to expression patterns of surface molecules, characterizes each phenotype.

Monocytes and macrophages are mononuclear phagocytes with crucial and distinct roles in transplant immunity. Monocytes display a remarkable plasticity in response to signals from the microenvironment, enabling them to differentiate into various cell types. Several pro-inflammatory, metabolic, and immune stimuli all increase the attraction of monocytes toward tissue (7). Based on the expression of CD14 (LPS co-receptor) and CD16 (Fcγ receptor III), three phenotypically and functionally distinct human monocyte subsets: CD14^++^CD16^−^ (classical), CD14^++^CD16^+^ (intermediate), and CD14^+^CD16^++^ (non-classical) monocytes can be defined (60–63). Monocytes arise from myeloid precursor cells in primary and secondary lymphoid organs, such as liver and bone marrow. In humans, monocytes represent, respectively, 10% of the nucleated cells in peripheral blood, with two major reservoirs: the spleen and lungs that can mobilize monocytes on demand (64, 65). Classical monocytes are able to start proliferating in the bone marrow in response to infection or tissue damage and subsequently be released into the circulation in a CCR2-dependent manner (Figure 2) (66). Intermediate and non-classical monocytes are thought to be descendants of classical monocytes that have been under control of transcription factor Nur77 (NR4A1) returned to the bone marrow (67). Non-classical monocytes show a patrolling, distinct motility and crawling pattern (68). Interestingly, intermediate monocytes show higher expression of major histocompatibility complex (MHC) class II molecules and thereby more related to non-classical monocytes (69, 70). CD14^+^ monocytes can be recruited to the site of inflammation or areas of tissue injury where they can differentiate into macrophages and dendritic cells (71). In steady state, circulating monocytes have minimal contribution to the maintenance of tissue-resident macrophages (72, 73). Depending on the microenvironment, activation stimuli, and cross talk with other immunological effector cells, activation of macrophages alters their cytokine profile and costimulatory molecule expression. Monocyte differentiation to tissue macrophages is colony-stimulating factor 1 receptor dependent. Most tissue macrophages are seeded before birth in embryonic state, with varying contributions of primitive-derived and definitive-derived cells. Monocytic input to tissue macrophage compartments seems to be restricted to inflammatory settings, such as infection and acute graft rejection (71). Monocyte chemotactic peptide 1 (MCP-1) is an important regulator of macrophage recruitment and was shown to be highly expressed in the kidney allograft, supporting the concept of recruitment of monocytes from the circulation (74).

Macrophages can be subdivided into “classically activated” or “alternatively activated.” Classically activated macrophages are described as M1 macrophages, which are developed upon response to IFNγ, LPS, or TNF-α. M1 macrophages express surface markers: MHCII, CD40, CD80, CD86, and CD11b. They can produce inflammatory cytokines such as TNF-α, IL-1, IL-6, IL-8, IL-12, CCL2, CXCL9, and CXCL10. M1 macrophages are linked to the Th1 response and are mainly considered as pro-inflammatory macrophages whereas M2 are considered as mainly anti-inflammatory. M2 macrophages can be subdivided into M2a, M2b, and M2c. M2a macrophages are generated on response to IL-4 and IL-13. Immune complexes and toll-like receptor (TLR)/IL-1R ligands activate M2b macrophages, whereas M2c macrophages are activated by IL-10, TGF-β, and glucocorticoids. M2 macrophages express surface markers: CD163, CD206, and CD209. M2 macrophages produce IL-10 and TGF-β mainly leading to tissue repair and scar formation. M2 macrophages are linked to Th2 response and show immune-modulatory functions (7, 71, 75). Human regulatory macrophages (Mregs) are in a specific state of differentiation with a robust phenotype and potent T-cell suppressor function. These Mregs arise from CD14^+^ peripheral blood monocytes during 7-day culture exposed to M-CSF and activation by IFNγ (76). Mregs express several molecules such as MHCII, FCγR, IFNγR, TLR-4, and PD-L1 as shown in Figure 2 (77). Shifting the balance between regulatory macrophages and/or monocytes on the one hand and the effector macrophages and pro-inflammatory monocytes on the other hand could theoretically result in dampening the immune response against the graft and the immunological tolerance, or to aggravation of graft rejection. To date, two clinical trials investigated the feasibility of regulatory macrophages in promoting allograft acceptance with promising results (78, 79). Moreover, recently, a new homogeneous monocyte subpopulation of human G-CSF-induced CD34^+^ monocytes with powerful immunosuppressive properties upon human allogeneic T-cell activation was described. Such tolerogenic monocytes could be used for novel immune regulatory or cellular therapy development (80).

Recently, an adaptive feature of innate immunity has been described as “trained immunity.” Trained immunity is defined as a non-specific immunological memory resulting from rewiring the epigenetic program and the functional state of the innate immunity (81). Twenty naïve patients were vaccinated for Bacille Calmette–Guérin (BCG) to investigate mechanisms of the enhanced immune function. Interestingly, these authors identified trained monocytes in the circulation of BCG-vaccinated individuals for at least 3 months suggesting that reprogramming takes place at the level of progenitor cells in the bone marrow (82). Recent evidence emerged to indicate that innate immune memory could be transferred *via* hematopoietic stem and progenitor cells. *In vitro* studies showed effects lasting for days (83, 84), whereas other reports showed memory effects for weeks (85). These interesting observations might be explained by alterations in epigenetic (de)methylation profiles after antigenic stimulation. Altering the epigenetic program by pharmacological means leading to behavioral changes of monocytes could be a promising method to restore or modify the healthy gene/protein expression in the pro-inflammatory microenvironment. The phenomenon of trained immunity in alloreactivity and transplantation may be a very interesting area of future research, i.e., innate memory toward donor antigens resulting from cross-reactivity with other microbial and/or viral agents.

## Rabbit Anti-Thymocyte Globulin (rATG) and Basiliximab and Monocyte/Macrophage Cell Lineage

Rabbit antithymocyte globulin is a polyclonal antibody with mainly T cell-depleting capacities. rATG can also induce B cell apoptosis and stimulate Treg and NKT cell generation (86). After rATG treatment, cytokine-dependent homeostatic proliferation of T cells is initiated (87). Basiliximab (anti-CD25 monoclonal antibody) blocks the CD25 receptor on the surface of activated T cells. Studies on the effects of basiliximab or rATG on monocytes/macrophages are scarce. However, one report showed a reduction in the percentage of CD14^+^CD16^+^ monocytes when PBMC were cultured *in vitro* in the presence of rATG (28). By contrast, this cell type was not affected by basiliximab, although low expression levels of CD25 on stimulated monocytes and macrophages are described (88, 89). These authors also reported a reduction of circulating CD14^+^CD16^+^ monocytes in kidney transplant patients treated with rATG during the first week after transplantation, while this was not seen for basiliximab induction therapy. Another part of the same study showed an upregulation of the percentage of CD14^+^CD163^+^ monocytes in either basiliximab- or rATG-treated kidney transplant recipients, which could be detected for a longer time period in the circulation than in patients without induction therapy. CD14^+^CD163^+^ monocytes are precursors for M2 macrophages and these cells are well known for their anti-inflammatory effect, suggesting that the upregulation of CD14^+^CD163^+^ cells may contribute to a better outcome after transplantation. However, this study only described the changes in the CD14^+^CD16^+^ monocyte subset after rATG or basiliximab therapy, while the effect on other subsets such as the classical CD14^++^CD16^−^ monocytes remains unknown. Therefore, it is unclear whether the pro-inflammatory immune response by monocytes is changed in the presence of rATG or basiliximab.

## Alemtuzumab and Monocyte/Macrophage Cell Lineage

The humanized monoclonal antibody alemtuzumab targets the CD52 molecule which is expressed at different levels on B cells, T cells, NK cells, dendritic cells, and monocytes. The CD52 molecule, also known as CAMPATH-1 antigen, is a glycoprotein of which the precise function is unclear, although it might be involved in T-cell migration and costimulation (90). However, monocytes are known to be less sensitive for the depleting effects of alemtuzumab than lymphocytes, despite their high CD52 expression (29–32). For example, in ACR dominated by monocytes, alemtuzumab treatment did not show depletion of monocytes in tissue, confirming the low sensitivity of monocytes to alemtuzumab treatment (91). An explanation for this low susceptibility could be the high expression levels of complement inhibitory proteins, which protect monocytes from complement mediated lysis (32). Another study showed repopulation of monocytes within 3 months after alemtuzumab therapy, while the recovery of T and B cells takes usually more than 1 year. Consequently, the low susceptibility of monocytes for alemtuzumab is thought to be one of the reasons for renal graft dysfunction after induction therapy with alemtuzumab, such as reperfusion and rejection (92). So far, this low susceptibility of monocytes to alemtuzumab therapy could be partially explained by the high expression of complementary inhibitory proteins that protect monocytes from getting lysed after alemtuzumab treatment (32). After alemtuzumab treatment, tissue monocytes in the rejecting graft showed an increased expression of the costimulatory molecules CD80 and CD86, a higher intracellular expression of NF-κB, and stronger production of IL-6 compared to patients without alemtuzumab therapy (30). Moreover, this pro-inflammatory cytokine production could facilitate kidney allograft rejection after alemtuzumab therapy, although other cell types, such as NK cells, could also contribute to rejection processes after alemtuzumab therapy (93).

## Calcineurin Inhibitors (CNIs) and Monocyte/Macrophage Cell Lineage

Tacrolimus and cyclosporine A inhibit the calcineurin pathway in T cells, which is also present in other cell types. As a consequence, the activation of the nuclear factor of activated T cells (NFAT) is blocked, leading to a reduced production of IL-2 and IFN-γ by T cells (94, 95). CNIs also have an effect on the mitogen-activated protein kinase (MAPK) signaling pathway *via* the inhibition of p38MAPK phosphorylation and consequently, reduced production of cytokines, such as IL-2, IL-10, TNF-α, and IFN-γ (96). The calcineurin and MAPK pathway are also present in macrophages, although the inhibitory effects of CNIs on T cells and macrophages are different (97). In more detail, tacrolimus was found to have no inhibitory effect on p38MAPK phosphorylation at low (5 ng/ml) and high (50 ng/ml) concentrations in LPS-activated monocytic THP-1 leukemia cells (50). However, another member of the MAPK pathway, extracellular signal-regulated kinase (ERK), did show less phosphorylation in the presence of a high concentration (50 ng/ml) of tacrolimus in monocytes as measured by western blotting, leading to a lower production of TNF-α. Kang et al. reported that monocyte signaling pathways were activated instead of inhibited by CNI *via* the inhibition of the calcineurin pathway and, as a consequence, the activation of the NF-κB signaling pathway (97). However, the concentrations of CNIs used in this study were supratherapeutic. Therefore, the observed induction in cytokine production, shown in this study, could also be explained by toxic lysis of the monocytes (34). Overall, these studies suggest that CNIs cannot suppress the activation of monocytes to the same degree as in T-cells.

Recognition of damage-associated molecular patterns by TLRs on the surface of monocytes leads to the activation of these cells and plays an important pathogenic role during transplant rejection (98–100). Both tacrolimus and cyclosporine can inhibit TLR signaling of PBMC in liver transplant patients, as shown by decreased production of IL-6 and TNF-α after TLR stimulation (34). CNIs act differently in suppressing the cytokine production upon TLR activation. For example, cyclosporine inhibits the production of TNF-α mediated by TLR7/8 and the production of IL-6 mediated by TLR2 and TLR7/8 signaling significantly more than tacrolimus (34). Moreover, monocytes from renal transplant recipients treated with tacrolimus showed an increased production of IL-1β, TNF-α, IL-6, and IL-10 after stimulation with LPS, in comparison to cyclosporine treated patients (101). Thus, the effect of CNIs on monocytes differs between tacrolimus and cyclosporine.

The different outcomes of tacrolimus and cyclosporine on cytokine production concerns only one of the monocyte/macrophage functions. Bacterial infections can have a significant impact on the graft after transplantation. Cyclosporine inhibits the phagocytosis of bacteria by macrophages *via* the alteration of nucleotide-binding oligomerization domain (NOD)-1 expression. The NOD-1 expression depends on the activation of the transcription factor NFAT, which is the main target of CNI (35). Thus, cyclosporine can promote bacterial infections after transplantation by altering phagocytic capacity of macrophages more rigorously.

## Mycophenolate Mofetil (MMF) and Monocyte/Macrophage Cell Lineage

Mycophenolate mofetil has led to significantly reduced rejection rate as compared to its counterpart azathioprine (102–104). The active metabolite mycophenolic acid reduces the synthesis of guanosine nucleotides *via* the inhibition of inosine monophosphate dehydrogenase, which is a more specific metabolic pathway for T and B cells than for other cell types (36, 105).

Circulating monocytes of kidney transplant recipients suffering from chronic rejection who were treated with MMF showed a decreased capacity to produce IL-1β, IL-10, and TNF-α as compared to circulating monocytes of chronic rejection patients who were not treated with MMF. Cytokine production capacity was measured by flow cytometry and confirmed by PCR on gene expression level (37). Moreover, the expression of the TNF-receptor 1 was decreased in the MMF treated group, suggesting a favorable effect in patients with chronic rejection (37). Furthermore, MMF reduced the expression of the adhesion molecules; intercellular adhesion molecule-1, and MHC II on isolated human monocytes (106).

## Glucocorticoids and Monocyte/Macrophage Cell Lineage

The immunosuppressive and anti-inflammatory effects of glucocorticoids are redundant and cover different stages of alloreactivity triggered by activation of donor-specific T cells after transplantation. Steroids can bind *via* passive diffusion to the intracellular glucocorticoid receptor. After translocation to the nucleus, steroids bind to the glucocorticoid response elements that have a connection with promoters of different genes. The anti-inflammatory effect of glucocorticoids is based on the transrepression of inflammatory gene transcription, such as the inhibition of the transcription factors AP-1 and NF-κB, and the transactivation of anti-inflammatory genes, including tyrosine aminotransferase and the induction of IκB (107–110). In this way, glucocorticoids control antigen presentation, cytokine production, and proliferation of lymphocytes.

In monocytes, glucocorticoids specially affect the heterogeneity of monocyte subsets (38, 111, 112). Flow cytometric analysis revealed that steroid treatment of stable kidney transplant patients for more than 12 months is associated with an increased absolute number of CD14^++^CD16^−^ and CD14^++^CD16^+^ monocyte subsets compared to patients without steroid intake. As a consequence, the counts for the non-classical CD14^+^CD16^++^ monocyte subset were significantly lower (38). Furthermore, glucocorticoids inhibit the upregulation of B7 molecules on the surface of human monocytes, which can negatively affect the antigen-presenting function of the cell (39, 113). The B7 family consists of many peripheral membrane proteins, including CD80 and CD86, which are all involved in the costimulatory signal needed for T cell activation. This suggests that glucocorticoid therapy in combination with belatacept therapy (blocking CD80/CD86) could theoretically block the immune response by T cells induced *via* antigen-presenting monocytes after transplantation.

The production of the anti-inflammatory cytokine IL-10 by monocytes is increased under treatment with methylprednisolone, while the production of the pro-inflammatory cytokines IL-12, IL-1, and TNF-α are downregulated in the presence of glucocorticoids (40, 41). Addition of 16 µg/ml of glucocorticoids *in vitro* leads to a decreased uptake of bacteria by monocytes, indicating that the phagocytosis of bacteria by monocytes is downregulated (42). Glucocorticoids are also known to drive the polarization of macrophages to a M2 phenotype (75, 114). This indicates that glucocorticoids drive the cytokine production by monocyte to a more anti-inflammatory phenotype and inhibits the phagocytic function of monocytes. Glucocorticoids enhance the uptake of apoptotic cells by macrophages trough the induction of Mer-Tk (MER proto-oncogene tyrosine kinase), thereby inducing macrophage reprogramming toward a regulatory phenotype, also called Meff, for macrophages performing efferocytosis (115–117). This approach has been evaluated in the treatment of collagen-induced arthritis (118), as well as acute graft rejection (119), justifying further exploration in the field of transplantation.

## Inhibitors of the Mammalian Target of Rapamycin (mTOR) and Monocyte/Macrophage Cell Lineage

The mTOR signaling pathway is involved in the activation, proliferation, differentiation, and translocation of T cells. Inhibitors of mTOR, such as everolimus and sirolimus, are therefore very useful after transplantation (120). The same mTOR inhibitors do also have an inhibitory effect on human monocytes by suppressing the production of the chemokines MCP-1, RANTES, IL-8, MIP-1α, and MIP-1β (43). Furthermore, the downstream effects of rapamycin therapy are characterized by a decreased production of the monocyte-derived cytokine IL-6 and an increase of TGF-beta production in comparison to MMF, as it was shown by fine-needle biopsy cultures from kidney transplant patients treated with either a cyclosporine–rapamycin–prednisone or a cyclosporine–MMF–prednisone therapy 1 week after transplantation (44). This resulted in a more tolerogenic effect of the monocytes and less graft rejection during the first 6 months after transplantation in comparison to a MMF-based drug therapy. Moreover, combined therapy of mTOR inhibitors and glucocorticoid therapy increased the production of the pro-inflammatory cytokines IL-12, TNF-α, and IL-1β (45). Altogether, mTOR inhibitors can inhibit cytokine production by monocytes shortly after transplantation, although a combination therapy with prednisone should be regarded with caution.

## Belatacept and Monocyte/Macrophage Cell Lineage

Belatacept, a fusion protein consisting of the extracellular domain of the human cytotoxic T-lymphocyte antigen (CTLA)-4 antigen linked to a Fc-fragment of immunoglobulin G1, inhibits the costimulatory signal between the CD80/CD86 molecules on antigen-presenting cells and the CD28 molecule on T cells, thereby preventing T cell activation (121). Monocytes express CD80/CD86 molecules, and, as a consequence, the antigen-presenting function of monocytes is blocked by belatacept (46, 122, 123). This suggests that belatacept inhibits the antigen-presenting function of monocytes/macrophages. In one case of acute rejection within 3 months after transplantation, the blockade of CD80/CD86 was incomplete under belatacept treatment, suggesting the importance of higher belatacept tissue concentrations needed to completely block monocyte antigen presentation function (123). Thus, belatacept, in controlled dosages, blocks the expression of CD80/CD86 on monocytes, thereby inhibiting their antigen-presenting function and activation of T cells.

The older variant of belatacept, abatacept (CTLA-4Ig), is frequently used in the treatment of patients with rheumatoid arthritis (RA) (47). After treatment with abatacept, the number of circulating monocytes was increased, and the phenotype of these cells was significantly changed, due to downregulation of actin fibers. For example, the capability of monocyte migration was negatively changed even as the number of adhesion molecules *in vitro*. Data were verified with monocytes from healthy controls. The reduced number of adhesion molecules and migration capacity could be a reason for the increased number of monocytes in the peripheral blood that cannot pass endothelial barriers, whereby it is no longer possible for the monocyte to contribute in inflammation.

Binding of abatacept to the CD80/CD86 receptor on macrophages from healthy blood donors is associated with decreased production of the pro-inflammatory cytokines IL-12 and TNF-α, suggesting again a role for abatacept/belatacept in changing the pro-inflammatory environment *via* macrophages after transplantation (48).

## Other Experimental Drugs and Monocyte/Macrophage Cell Lineage

Although no monocyte specific drugs as such exist now, multiple experimental and less known drugs do influence monocyte functions. Looking outside the box of currently used immunosuppressive drugs in SOT, there are a few compounds with immune-inhibitory effects, which theoretically could be interesting in combating alloimmunity. For example, the human monoclonal antibody canakinumab, originally designed as an interleukin-beta (IL-1β) inhibitor for the repression of inflammation in autoimmune diseases, can also inhibit the IL-1β production by monocytes (124). A high expression of IL-1β is noticed in the most severe liver transplant rejection episodes and at the time of kidney transplantation, suggesting the importance of blocking its production by monocytes (26, 49). However, treatment of kidney transplant recipients with canakinumab can inhibit IL-1β secretion in many other cell types, leading to undesirable side effects (125).

Infliximab, originally used in the treatment of autoimmune diseases, is another monoclonal antibody targeting monocyte TNF-α production. Monocytes and macrophages are main producers of TNF-α, suggesting the importance of infliximab for targeting monocytes (126). Beside the effect on TNF-α production, monocytes from Crohn’s disease patients treated with therapeutic concentrations of infliximab showed also increased apoptosis *via* the activation of caspase-3, 8, and 9 (127).

Furthermore, the herbal medicine sinomenine was found to reduce migration of activated human monocyte cells and inhibits human monocyte-derived DC differentiation and maturation (50, 51). In addition, peripheral blood monocytes from healthy donors cultured for 60 h in the presence of different concentrations of sinomenine showed an enhanced production of IL-6 and a decreased expression of IL-8, which is important for cell migration (128). This would suggest a positive effect of sinomenine on monocyte infiltration and migration, although there is still an increased production of pro-inflammatory cytokines. However, this research was performed using monocytic THP-1 cell-line, and isolated peripheral blood monocytes from healthy donors, so that possible effects with regard to transplantation are still unknown.

15-Deoxyspergualin or gusperimus is a relatively long-known immunosuppressive drug with an inhibitory effect on monocyte proliferation, TNF-α production, and phagocytotic functions of monocytes. More recently, it was been suggested that gusperimus can also be effective in suppressing the antigen presentation function of monocytes in transplantation (52). Another member of the spergualin family is LF15-0195. This drug is known for its inhibitory effect on monocyte accumulation in the tubulointerstitial compartment of rat kidneys and was shown to have beneficial effects in the treatment of glomerulonephritis (129).

In diabetes mellitus, macrophage accumulation and activation play a central role in disease progression. Research on simvastatin, a drug to lower elevated lipid levels, has been shown to effectively lower IL-6, IL-8, TNF cytokine, and superoxide anion production by monocytes isolated from human blood samples of patients with diabetes mellitus type 1 (59). In addition, simvastatin reduces the NF-κB activity in monocytes with approximately 60%, which causes the inhibition of IL-6 and IL-8 production. Treatment of IgA nephropathy with the drug atorvastatin showed a reduction of monocyte proliferation (130). In diabetes mellitus type 2 patients, this drug lowers the TNF-alpha production by monocytes (131). Other studies in diabetes mellitus patients have shown potential effects of salsalate on macrophages activation. Salsalate, a prodrug of salicylic acid, is also known for the inhibition of the NF-κB pathway in macrophages (53, 54). This suggests a working mechanism for salsalate that is similar to simvastatin. Both drugs can be promising compounds to inhibit monocyte and macrophage activation.

In RA, research on therapeutic drugs to target monocytes and macrophages is more common because of the important role of monocytes in developing this disease. In addition, TNF-α is a key player known to cause inflammation in RA and is mainly produced by monocytes (132). Some of the drugs used to suppress inflammation in RA could also have a potential in transplantation. For example, a decreased number of CD14^+^CD16^+^ monocytes were found after treatment of RA patients with tocilizumab, an IL-6 receptor blocker (133). In addition, production of IL-6 by monocytes from healthy donors was reduced when tocilizumab was added *in vitro*. The drug also induces the apoptosis of staphylococcal enterotoxin B-activated monocytes (55). These results suggest that tocilizumab could theoretically impair the monocyte responses after transplantation. Furthermore, bromodomain and extra-terminal (BET) inhibitors are developed to control the intracellular chromatin regulation responsible for the activation of monocytes, thereby inhibiting inflammation processes induced by monocytes. In more detail, CD14^+^ monocytes were isolated from blood samples of healthy volunteers and cultured in the presence of BET inhibitors and IFN-β, IFN-γ, IL-4, and IL-10 stimuli, where after the intracellular activation cytokine response were suppressed (56). In RA patients, this epigenetic control by BET inhibitors could suppress the production of pro-inflammatory cytokines and chemokines such as CXCL10. This would indicate that BET inhibitors could also inhibit monocyte activation after transplantation, although this is very speculative and require more research.

Fish oil-based drugs, such as lovaza, are used to lower triglyceride levels in obesity. These fish oil compounds demonstrated a reduction in the number of macrophages and reduced MCP-1 blood levels (57). Eicosapentaenoic acid, one of the major fatty acids in fish oil, reduces the secretion of TNF-α by human monocytic THP-1 cells, *via* the inhibition of the intracellular NF-κB activation (58). This suggests also a suppressing role for fatty acids in monocyte activation that could have a potential effect in transplantation as well.

## Future Challenges and Developments

**Figure 3:**
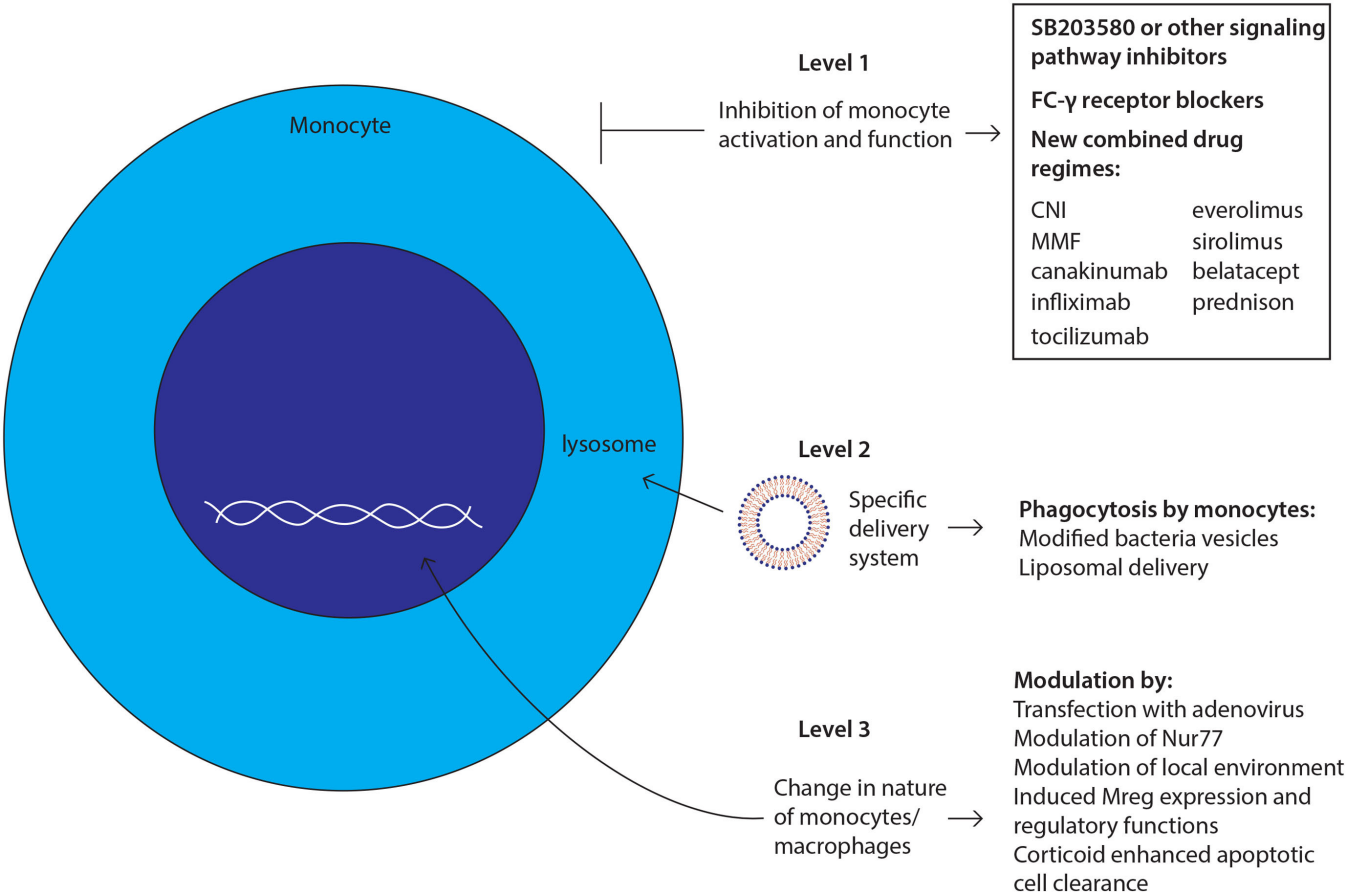
**Future challenges and developments: strategies to target monocytes/macrophages**. New therapies targeting monocytes and macrophages could intervene at three levels with monocyte actions and their subsequent functions as depicted and described in manuscript body.

Therapies targeting monocytes and macrophages in (SOT) could intervene at different points with monocyte actions and their subsequent functions (Figure 3). First, the activation and function of the cells can be inhibited at multiple stages: signaling pathway activation, antigen presentation, and cytokine production. Blockade of the intracellular signaling pathways inhibits the activation of monocytes and macrophages. For example, the use of specific MAPK inhibitors, such as SB203580, blocks the activation of monocytes (134). However, these drugs will also block the activation of many other cell types. Targeting antigen presentation is even more difficult than targeting signaling pathways. It is known that the Fcγ receptor on monocytes is involved in the recognition and processing of donor antigen-specific antibodies (135, 136). Blocking this receptor with specific antibodies could inhibit the antigen presentation function of monocytes. Furthermore, already existing drugs that reduce the cytokine production by monocytes and macrophages, for example, canakinumab, infliximab, and tocilizumab, mainly target the inhibition of one single cytokine. To be more effective, monocyte-specific drugs should be developed to inhibit the production or the effects of multiple cytokines at once, thereby reducing side effects.

Second, delivering any potential new drug to the target cell, in this case monocytes and macrophages, is a major point of intervention, which could lower the side effects. One can envision a delivery system using the phagocytosis function of the monocyte/macrophage, whereby macrophages can ingest immunosuppressive drug-loaded-inactivated bacteria or liposomes carrying the potential new drug (137). However, the monocyte is not the only cell type with a phagocytic system. Therefore, the surface of these bacteria or liposomes should be modified to facilitate the specific recognition by the monocyte/macrophage in order to overcome side effects. Another approach to target monocytes and macrophages *via* their phagocytotic function is to use apoptotic cells through a process that is known as efferocytosis (116, 117). Phagocytosis of these apoptotic cells by monocytes and macrophages will induce an anti-inflammatory response at the tissue level and may induce immunological tolerance. Furthermore, *ex vivo* experiments showed a decrease in CD11b expression on macrophages (138), suggesting that treatment with apoptotic cells induces the generation of Mregs. As mentioned above (see Glucocorticoids and Monocyte/Macrophage Cell Lineage), the uptake of apoptotic cells can be enhanced by treatment with glucocorticoids (116).

The third point of therapeutic efficacy would be the manipulation of the nature of these cells. The future of *in vivo* manipulation of macrophages is intriguing; phenotypes could be changed by transfection with adenovirus, modulation of nuclear transcription factor NR4A1 (Nur77), or by modulation of local microenvironment with cytokines to polarize macrophages to reparative phenotype (67). Targeting all monocytes and macrophages indiscriminately could also be a disadvantage as regulatory and effector macrophages also have beneficial effects including the control of infections and the induction of regulatory cells (139).

Moreover, inhibition of all macrophages will also affect the number of Mregs, which are important for inducing tolerance after transplantation (140). Too much inhibition of effector macrophages or Mregs could lead to graft rejection or complications, such as atherosclerosis and cardiovascular diseases. Furthermore, currently prescribed immunosuppressive drugs might miss the power to upregulate Mregs efficiently. In experimental mouse models, Mregs have demonstrated anti-inflammatory and T-cell-suppressing effects (other beneficial effects of Mregs are described in Section “Monocyte Immunobiology”) (141, 142). A more specific upregulation of these cells could be an approach to beneficially shift the balance toward macrophages controlling immune responses including those in organ transplant patients. Ideally, after SOT, the balance of macrophage subsets should be in favor of macrophages that control the anti-donor response, while the accumulation of macrophages with pro-inflammatory and antigen presentation characteristics should be decreased (143, 144). For example, reduced function of the detrimental functions of macrophages involved in alloreactivity might be a useful therapy, although more research is needed to find a specific approach. Another way to differentiate between effector and controlling functions of macrophages could be by polarizing cells into M1 and M2 subsets. Targeting specific signaling pathways involved in this polarization process such as the Notch signaling pathway could change the nature of these cells to a more anti-inflammatory phenotype (145). NF-κB signaling, controlled by the Notch pathway, is associated with pro-inflammatory macrophage responses, while a more anti-inflammatory phenotype is induced *via* the ERK pathway (145, 146). Targeting these pathways with specific stimuli may change the phenotype of macrophages. Stimuli that induce macrophage polarization toward a M1 phenotype are GM-CSF, IFN-γ, and LPS, while IL-4, IL-13, and IL-10 enhance a M2 macrophage phenotype (147). Future insight and research are necessary to investigate the effect of these manipulated macrophages on healthy and diseased tissue.

Ideally, a potential new drug inhibiting monocytes–macrophages at these three levels would change the spectrum of not only rejection treatment or prevention after (SOT) but also the course of many autoimmune mediated diseases. Either alone or in combination with other existing immunosuppressive drugs, this field constitutes a challenging area of future therapeutic research.

## Author Contributions

NK and TB contributed in the process of writing/design and discussing; DH contributed in the process of discussion and reviewing; CB and AR contributed in the process of writing/design/discussing and reviewing.

## Conflict of Interest Statement

The authors declare that the research was conducted in the absence of any commercial or financial relationships that could be construed as a potential conflict of interest.

## Abbreviations

ATG, antithymocyte globulin; BET, bromodomain and extra-terminal; CNI, calcineurin inhibitor; CTLA-4, Ig cytotoxic T-lymphocyte-associated protein 4 immunoglobulin; ERK, extracellular signal-regulated kinase; GRE, glucocorticoid response element; ICAM, intercellular adhesion molecule; MAPK, mitogen-activated protein kinase; MCP, monocyte chemoattractant protein; MHC, major histocompatibility complex; MMF, mycophenolate mofetil; MPA, mycophenolic acid; NF-κB, nuclear factor kappa B; NFAT, nuclear factor of activated T cells; NOD, nucleotide-binding oligomerization domain; RA, rheumatoid arthritis; SEB, staphylococcal enterotoxin B; TAT, tyrosine aminotransferase; TLR, toll-like receptor.

## References

[1] Koo EH Jang HR Lee JE Park JB Kim S-J Kim DJ The impact of early and late acute rejection on graft survival in renal transplantation. Kidney Res Clin Pract (2015) 34(3):160–4. 10.1016/j.krcp.2015.06.003 26484041 PMC4608868

[2] Einecke G Sis B Reeve J Mengel M Campbell PM Hidalgo LG Antibody-mediated microcirculation injury is the major cause of late kidney transplant failure. Am J Transplant (2009) 9(11):2520–31. 10.1111/j.1600-6143.2009.02799.x 19843030

[3] Sellares J de Freitas DG Mengel M Reeve J Einecke G Sis B Understanding the causes of kidney transplant failure: the dominant role of antibody-mediated rejection and nonadherence. Am J Transplant (2012) 12(2):388–99. 10.1111/j.1600-6143.2011.03840.x 22081892

[4] Meier-Kriesche HU Schold JD Srinivas TR Kaplan B. Lack of improvement in renal allograft survival despite a marked decrease in acute rejection rates over the most recent era. Am J Transplant (2004) 4(3):378–83. 10.1111/j.1600-6143.2004.00332.x 14961990

[5] Nankivell BJ Alexander SI Rejection of the kidney allograft. N Engl J Med (2010) 363(15):1451–62. 10.1056/NEJMra0902927 20925547

[6] Mannon RB Macrophages: contributors to allograft dysfunction, repair or innocent bystanders? Curr Opin Organ Transplant (2012) 17(1):20–5. 10.1097/MOT.0b013e32834ee5b6 22157320 PMC3319132

[7] Rowshani AT Vereyken EJF. The role of macrophage lineage cells in kidney graft rejection and survival. Transplantation (2012) 94(4):309–18. 10.1097/TP.0b013e318250c10f 22828735

[8] van Kooten C Daha MR. Cytokine cross-talk between tubular epithelial cells and interstitial immunocompetent cells. Curr Opin Nephrol Hypertens (2001) 10(1):55–9. 10.1097/00041552-200101000-00009 11195052

[9] Girlanda R Kleiner DE Duan Z Ford EAS Wright EC Mannon RB Monocyte infiltration and kidney allograft dysfunction during acute rejection. Am J Transplant (2008) 8(3):600. 10.1111/j.1600-6143.2007.02109.x 18294156 PMC2813043

[10] Moreau A Varey E Anegon I Cuturi MC Effector mechanisms of rejection. Cold Spring Harb Perspect Med (2013) 3(11):1–33. 10.1101/cshperspect.a015461 PMC3808773 24186491

[11] Om A Baquero A Raja R Kim P Bannett AD The prognostic significance of the presence of monocytes in glomeruli of renal transplant allografts. Transplant Proc (1987) 19(1):1618–22. 3547871

[12] Copin MC Noel C Hazzan M Janin A Pruvot FR Dessaint JP Diagnostic and predictive value of an immunohistochemical profile in asymptomatic acute rejection of renal allografts. Transpl Immunol (1995) 3(3):229–39. 10.1016/0966-3274(95)80029-8 8581411

[13] Grimm PC McKenna R Nickerson P Russell ME Gough JIM Gospodarek E Clinical rejection is distinguished from subclinical rejection by increased infiltration by a population of activated macrophages. Clin J Am Soc Nephrol (1999) 10(7):1582–9. 10405215 10.1681/ASN.V1071582

[14] Özdemir BH Demirhan B Güngen Y. The presence and prognostic importance of glomerular macrophage infiltration in renal allografts. Nephron (2002) 90(4):442–6. 10.1159/000054732 11961403

[15] Tinckam KJ Djurdjev O Magil AB. Glomerular monocytes predict worse outcomes after acute renal allograft rejection independent of C4d status. Kidney Int (2005) 68(4):1866–74. 10.1111/j.1523-1755.2005.00606.x 16164665

[16] Fahim T Böhmig GA Exner M Huttary N Kerschner H Kandutsch S The cellular lesion of humoral rejection: predominant recruitment of monocytes to peritubular and glomerular capillaries. Am J Transplant (2007) 7(2):385–93. 10.1111/j.1600-6143.2006.01634.x 17283488

[17] Kozakowski N Böhmig GA Exner M Soleiman A Huttary N Nagy-Bojarszky K Monocytes/macrophages in kidney allograft intimal arteritis: no association with markers of humoral rejection or with inferior outcome. Nephrol Dial Transplant (2009) 24(6):1979–86. 10.1093/ndt/gfp045 19223275 PMC2997283

[18] Kwan T Wu H Chadban SJ. Macrophages in renal transplantation: roles and therapeutic implications. Cell Immunol (2014) 291(1–2):58–64. 10.1016/j.cellimm.2014.05.009 24973994

[19] Toki D Zhang W Hor KLM Liuwantara D Alexander SI Yi Z The role of macrophages in the development of human renal allograft fibrosis in the first year after transplantation. Am J Transplant (2014) 14(9):2126–36. 10.1111/ajt.12803 25307039

[20] Sentís A Kers J Yapici U Claessen N Roelofs JJTH Bemelman FJ The prognostic significance of glomerular infiltrating leukocytes during acute renal allograft rejection. Transpl Immunol (2015) 33(3):168–75. 10.1016/j.trim.2015.10.004 26494157

[21] Bergler T Jung B Bourier F Kühne L Banas MC Rümmele P Infiltration of macrophages correlates with severity of allograft rejection and outcome in human kidney transplantation. PLoS One (2016) 11(6):e0156900. 10.1371/journal.pone.0156900 27285579 PMC4902310

[22] Xu L Collins J Drachenberg C KuKuruga D Burke A. Increased macrophage density of cardiac allograft biopsies is associated with antibody-mediated rejection and alloantibodies to HLA antigens. Clin Transplant (2014) 28(5):554–60. 10.1111/ctr.12348 24580037

[23] Fishbein GA Fishbein MC. Morphologic and immunohistochemical findings in antibody-mediated rejection of the cardiac allograft. Hum Immunol (2012) 73(12):1213–7. 10.1016/j.humimm.2012.07.011 22813651

[24] Oberbarnscheidt MH Zeng Q Li Q Dai H Williams AL Shlomchik WD Non-self recognition by monocytes initiates allograft rejection. J Clin Invest (2014) 124(8):3579–89. 10.1172/JCI74370 24983319 PMC4109551

[25] Boersema M van den Born JC van Ark J Harms G Seelen MA van Dijk MC CD16+ monocytes with smooth muscle cell characteristics are reduced in human renal chronic transplant dysfunction. Immunobiology (2015) 220(5):673–83. 10.1016/j.imbio.2014.11.011 25476849

[26] Vereyken EJ Kraaij MD Baan CC Rezaee F Weimar W Wood KJ A shift towards pro-inflammatory CD16+ monocyte subsets with preserved cytokine production potential after kidney transplantation. PLoS One (2013) 8(7):e70152 10.1371/journal.pone.0070152 23922945 PMC3726371

[27] Kraaij MD Vereyken EJF Leenen PJM van den Bosch TPP Rezaee F Betjes MGH Human monocytes produce interferon-gamma upon stimulation with LPS. Cytokine (2014) 67(1):7–12. 10.1016/j.cyto.2014.02.001 24680476

[28] Sekerkova A Krepsova E Brabcova E Slatinska J Viklicky O Lanska V CD14+CD16+ and CD14+CD163+ monocyte subpopulations in kidney allograft transplantation. BMC Immunol (2014) 15:4. 10.1186/1471-2172-15-4 24499053 PMC3918100

[29] Hale G Xia M-Q Tighe HP Dyer MJS Waldmann H The CAMPATH-1 antigen (CDw52). Tissue Antigens (1990) 35(3):118–27. 10.1111/j.1399-0039.1990.tb01767.x 2165283

[30] Kirk AD Hale DA Mannon RB Kleiner DE Hoffmann SC Kampen RL Results from a human renal allograft tolerance trial evaluating the humanized CD52-specific monoclonal antibody alemtuzumab (CAMPATH-1H). Transplantation (2003) 76(1):120–9. 10.1097/01.TP.0000071362.99021.D9 12865797

[31] Fabian I Flidel O Gadish M Kletter Y Slavin S Nagler A. Effects of CAMPATH-1 antibodies on the functional activity of monocytes and polymorphonuclear neutrophils. Exp Hematol (1993) 21(12):1522–7. 8405234

[32] Rao SP Sancho J Campos-Rivera J Boutin PM Severy PB Weeden T Human peripheral blood mononuclear cells exhibit heterogeneous CD52 expression levels and show differential sensitivity to alemtuzumab mediated cytolysis. PLoS One (2012) 7(6):e39416. 10.1371/journal.pone.0039416 22761788 PMC3382607

[33] Escolano A Martínez-Martínez S Alfranca A Urso K Izquierdo HM Delgado M Specific calcineurin targeting in macrophages confers resistance to inflammation via MKP-1 and p38. EMBO J (2014) 33(10):1117–33. 10.1002/embj.201386369 24596247 PMC4193919

[34] Howell J Sawhney R Testro A Skinner N Gow P Angus P Cyclosporine and tacrolimus have inhibitory effects on toll-like receptor signaling after liver transplantation. Liver Transpl (2013) 19(10):1099–107. 10.1002/lt.23712 23894100

[35] Tourneur E Ben Mkaddem S Chassin C Bens M Goujon J-M Charles N Cyclosporine a impairs nucleotide binding oligomerization domain (nod1)-mediated innate antibacterial renal defenses in mice and human transplant recipients. PLoS Pathog (2013) 9(1):e1003152. 10.1371/journal.ppat.1003152 23382681 PMC3561241

[36] Allison AC Eugui EM. Mycophenolate mofetil and its mechanisms of action. Immunopharmacology (2000) 47(2–3):85–118. 10.1016/S0162-3109(00)00188-0 10878285

[37] Weimer R Mytilineos J Feustel A Preiss A Daniel V Grimm H Mycophenolate mofetil-based immunosuppression and cytokine genotypes: effects on monokine secretion and antigen presentation in long-term renal transplant recipients. Transplantation (2003) 75(12):2090–9. 10.1097/01.TP.0000058808.37349.23 12829918

[38] Rogacev KS Zawada AM Hundsdorfer J Achenbach M Held G Fliser D Immunosuppression and monocyte subsets. Nephrol Dial Transplant (2015) 30(1):143–53. 10.1093/ndt/gfu315 25313167

[39] Girndt M Sester U Kaul H Hünger F Köhler H. Glucocorticoids inhibit activation-dependent expression of costimulatory molecule B7-1 in human monocytes. Transplantation (1998) 66(3):370–5. 10.1097/00007890-199808150-00015 9721807

[40] Hodge G Hodge S Reynolds PN Holmes M Up-regulation of interleukin-8, interleukin-10, monocyte chemotactic protein-1, and monocyte chemotactic protein-3 in peripheral blood monocytes in stable lung transplant recipients: are immunosuppression regimens working? Transplantation (2005) 79(4):387–91. 10.1097/01.TP.0000151631.66884.2E 15729163

[41] Blotta MH DeKruyff RH Umetsu DT. Corticosteroids inhibit IL-12 production in human monocytes and enhance their capacity to induce IL-4 synthesis in CD4+ lymphocytes. J Immunol (1997) 158(12):5589–95. 9190905

[42] Rinehart JJ Balcerzak SP Sagone AL LoBuglio AF. Effects of corticosteroids on human monocyte function. J Clin Invest (1974) 54(6):1337–43. 10.1172/JCI107880 4612058 PMC301688

[43] Lin HY-H Chang K-T Hung C-C Kuo C-H Hwang S-J Chen H-C Effects of the mTOR inhibitor rapamycin on monocyte-secreted chemokines. BMC Immunol (2014) 15(1):37. 10.1186/s12865-014-0037-0 25257976 PMC4189728

[44] Oliveira JGG Xavier P Sampaio SM Henriques C Tavares I Mendes AA Compared to mycophenolate mofetil, rapamycin induces significant changes on growth factors and growth factor receptors in the early days postkidney transplantation. Transplantation (2002) 73(6):915–20. 10.1097/00007890-200203270-00015 11923692

[45] Weichhart T Haidinger M Katholnig K Kopecky C Poglitsch M Lassnig C Inhibition of mTOR blocks the anti-inflammatory effects of glucocorticoids in myeloid immune cells. Blood (2011) 117(16):4273–83. 10.1182/blood-2010-09-310888 21368289

[46] Latek R Fleener C Lamian V Kulbokas E III Davis PM Suchard SJ Assessment of belatacept-mediated costimulation blockade through evaluation of CD80/86-receptor saturation. Transplantation (2009) 87(6):926–33. 10.1097/TP.0b013e31819b5a58 19300198

[47] Bonelli M Ferner E Goschl L Bluml S Hladik A Karonitsch T Abatacept (CTLA-4IG) treatment reduces the migratory capacity of monocytes in patients with rheumatoid arthritis. Arthritis Rheum (2013) 65(3):599–607. 10.1002/art.37787 23203906

[48] Wenink MH Santegoets KCM Platt AM van den Berg WB van Riel PL Garside P Abatacept modulates proinflammatory macrophage responses upon cytokine-activated T cell and Toll-like receptor ligand stimulation. Ann Rheum Dis (2011) 71(1):80–3. 10.1136/annrheumdis-2011-200348 21908454

[49] Hoffmann MW Wonigeit K Steinhoff G Herzbeck H Flad HD Pichlmayr R. Production of cytokines (TNF-alpha, IL-1-beta) and endothelial cell activation in human liver allograft rejection. Transplantation (1993) 55(2):329–35. 10.1097/00007890-199302000-00019 8094579

[50] Ou YQ Chen LH Li XJ Lin ZB Li WD. Sinomenine influences capacity for invasion and migration in activated human monocytic THP-1 cells by inhibiting the expression of MMP-2, MMP-9, and CD147. Acta Pharmacol Sin (2009) 30(4):435–41. 10.1038/aps.2009.21 19305422 PMC4002274

[51] Wang Q Li X-K. Immunosuppressive and anti-inflammatory activities of sinomenine. Int Immunopharmacol (2011) 11(3):373–6. 10.1016/j.intimp.2010.11.018 21109035

[52] Perenyei M Jayne DR Flossmann O. Gusperimus: immunological mechanism and clinical applications. Rheumatology (2014) 53(10):1732–41. 10.1093/rheumatology/ket451 24501242

[53] Donath MY Shoelson SE. Type 2 diabetes as an inflammatory disease. Nat Rev Immunol (2011) 11(2):98–107. 10.1038/nri2925 21233852

[54] McCarty MF. Salsalate may have broad utility in the prevention and treatment of vascular disorders and the metabolic syndrome. Med Hypotheses (2010) 75(3):276–81. 10.1016/j.mehy.2009.12.027 20080359

[55] Tono T Aihara S Hoshiyama T Arinuma Y Nagai T Hirohata S. Effects of anti-IL-6 receptor antibody on human monocytes. Mod Rheumatol (2015) 25(1):79–84. 10.3109/14397595.2014.914016 24842475

[56] Chan CH Fang C Qiao Y Yarilina A Prinjha RK Ivashkiv LB. BET bromodomain inhibition suppresses transcriptional responses to cytokine-Jak-STAT signaling in a gene-specific manner in human monocytes. Eur J Immunol (2015) 45(1):287–97. 10.1002/eji.201444862 25345375 PMC4293348

[57] Spencer M Finlin BS Unal R Zhu B Morris AJ Shipp LR Omega-3 fatty acids reduce adipose tissue macrophages in human subjects with insulin resistance. Diabetes (2013) 62(5):1709–17. 10.2337/db12-1042 23328126 PMC3636648

[58] Zhao Y Joshi-Barve S Barve S Chen LH. Eicosapentaenoic acid prevents LPS-induced TNF-alpha expression by preventing NF-kappaB activation. J Am Coll Nutr (2004) 23(1):71–8. 10.1080/07315724.2004.10719345 14963056

[59] Jialal I Miguelino E Griffen SC Devaraj S. Concomitant reduction of low-density lipoprotein-cholesterol and biomarkers of inflammation with low-dose simvastatin therapy in patients with type 1 diabetes. J Clin Endocrinol Metab (2007) 92(8):3136–40. 10.1210/jc.2007-0453 17519305 PMC2677961

[60] Wong KL Yeap WH Tai JJ Ong SM Dang TM Wong SC. The three human monocyte subsets: implications for health and disease. Immunol Res (2012) 53(1–3):41–57. 10.1007/s12026-012-8297-3 22430559

[61] Ziegler-Heitbrock L Hofer TP. Toward a refined definition of monocyte subsets. Front Immunol (2013) 4:23. 10.3389/fimmu.2013.00023 23382732 PMC3562996

[62] Ziegler-Heitbrock L. Monocyte subsets in man and other species. Cell Immunol (2014) 289(1–2):135–9. 10.1016/j.cellimm.2014.03.019 24791698

[63] Ziegler-Heitbrock L. Blood monocytes and their subsets: established features and open questions. Front Immunol (2015) 6:423. 10.3389/fimmu.2015.00423 26347746 PMC4538304

[64] van Furth R Sluiter W. Distribution of blood monocytes between a marginating and a circulating pool. J Exp Med (1986) 163(2):474–9. 10.1084/jem.163.2.474 3944542 PMC2188035

[65] Swirski FK Nahrendorf M Etzrodt M Wildgruber M Cortez-Retamozo V Panizzi P Identification of splenic reservoir monocytes and their deployment to inflammatory sites. Science (2009) 325(5940):612–6. 10.1126/science.1175202 19644120 PMC2803111

[66] Terry RL Miller SD Molecular control of monocyte development. Cell Immunol (2014) 291(1–2):16–21. 10.1016/j.cellimm.2014.02.008 24709055 PMC4162862

[67] Hanna RN Carlin LM Hubbeling HG Nackiewicz D Green AM Punt JA The transcription factor NR4A1 (Nur77) controls bone marrow differentiation and the survival of Ly6C- monocytes. Nat Immunol (2011) 12(8):778–85. 10.1038/ni.2063 21725321 PMC3324395

[68] Ghattas A Griffiths HR Devitt A Lip GYH Shantsila E. Monocytes in coronary artery disease and atherosclerosis: where are we now? J Am Coll Cardiol (2013) 62(17):1541–51. 10.1016/j.jacc.2013.07.043 23973684

[69] Brooks CF Moore M. Differential MHC class II expression on human peripheral blood monocytes and dendritic cells. Immunology (1988) 63(2):303–11. 3350576 PMC1454506

[70] Frankenberger M Hofer TPJ Marei A Dayyani F Schewe S Strasser C Transcript profiling of CD16-positive monocytes reveals a unique molecular fingerprint. Eur J Immunol (2012) 42(4):957–74. 10.1002/eji.201141907 22531920

[71] Ginhoux F Jung S. Monocytes and macrophages: developmental pathways and tissue homeostasis. Nat Rev Immunol (2014) 14(6):392–404. 10.1038/nri3671 24854589

[72] Hashimoto D Chow A Noizat C Teo P Beasley MB Leboeuf M Tissue-resident macrophages self-maintain locally throughout adult life with minimal contribution from circulating monocytes. Immunity (2013) 38(4):792–804. 10.1016/j.immuni.2013.04.004 23601688 PMC3853406

[73] Yona S Kim K-W Wolf Y Mildner A Varol D Breker M Fate mapping reveals origins and dynamics of monocytes and tissue macrophages under homeostasis. Immunity (2013) 38(1):79–91. 10.1016/j.immuni.2012.12.001 23273845 PMC3908543

[74] Prodjosudjadi W Daha MR Gerritsma JS Florijn KW Barendregt JN Bruijn JA Increased urinary excretion of monocyte chemoattractant protein-1 during acute renal allograft rejection. Nephrol Dial Transplant (1996) 11:7. 8671975

[75] Martinez FO Gordon S. The M1 and M2 paradigm of macrophage activation: time for reassessment. F1000Prime Rep (2014) 6:13. 10.12703/P6-13 24669294 PMC3944738

[76] Hutchinson JA Riquelme P Sawitzki B Tomiuk S Miqueu P Zuhayra M Cutting edge: immunological consequences and trafficking of human regulatory macrophages administered to renal transplant recipients. J Immunol (2011) 187(5):2072–8. 10.4049/jimmunol.1100762 21804023

[77] Fleming BD Mosser DM. Regulatory macrophages: setting the threshold for therapy. Eur J Immunol (2011) 41(9):2498–502. 10.1002/eji.201141717 21952805 PMC4299459

[78] Hutchinson JA Brem-Exner BG Riquelme P Roelen D Schulze M Ivens K A cell-based approach to the minimization of immunosuppression in renal transplantation. Transpl Int (2008) 21(8):742–54. 10.1111/j.1432-2277.2008.00692.x 18573141

[79] Hutchinson JA Riquelme P Brem-Exner BG Schulze M Matthäi M Renders L Transplant acceptance-inducing cells as an immune-conditioning therapy in renal transplantation. Transpl Int (2008) 21(8):728–41. 10.1111/j.1432-2277.2008.00680.x 18573142

[80] D’Aveni M Rossignol J Coman T Sivakumaran S Henderson S Manzo T G-CSF mobilizes CD34+ regulatory monocytes that inhibit graft-versus-host disease. Sci Transl Med (2015) 7(281):281ra42. 10.1126/scitranslmed.3010435 25834108

[81] Netea MG Joosten LAB Latz E Mills KHG Natoli G Stunnenberg HG Trained immunity: a program of innate immune memory in health and disease. Science (2016) 352(6284):aaf1098. 10.1126/science.aaf1098 27102489 PMC5087274

[82] Kleinnijenhuis J Quintin J Preijers F Joosten LAB Ifrim DC Saeed S Bacille Calmette-Guérin induces NOD2-dependent nonspecific protection from reinfection via epigenetic reprogramming of monocytes. Proc Natl Acad Sci U S A (2012) 109(43):17537–42. 10.1073/pnas.1202870109 22988082 PMC3491454

[83] Quintin J Saeed S Martens JH Giamarellos-Bourboulis EJ Ifrim DC Logie C *Candida albicans* infection affords protection against reinfection via functional reprogramming of monocytes. Cell Host Microbe (2012) 12(2):223–32. 10.1016/j.chom.2012.06.006 22901542 PMC3864037

[84] Ostuni R Piccolo V Barozzi I Polletti S Termanini A Bonifacio S Latent enhancers activated by stimulation in differentiated cells. Cell (2013) 152(1–2):157–71. 10.1016/j.cell.2012.12.018 23332752

[85] Yoshida K Ishii S Innate immune memory via ATF7-dependent epigenetic changes. Cell Cycle (2016) 15(1):3–4. 10.1080/15384101.2015.1112687 26556024 PMC4825762

[86] Mohty M. Mechanisms of action of antithymocyte globulin: T-cell depletion and beyond. Leukemia (2007) 21(7):1387–94. 10.1038/sj.leu.2404683 17410187

[87] Bouvy AP Kho MM Klepper M Litjens NH Betjes MG Weimar W Kinetics of homeostatic proliferation and thymopoiesis after rATG induction therapy in kidney transplant patients. Transplantation (2013) 96(10):904–13. 10.1097/TP.0b013e3182a203e4 23985721

[88] Valitutti S Carbone A Castellino F Maggiano N Ricci R Larocca LM The expression of functional IL-2 receptor on activated macrophages depends on the stimulus applied. Immunology (1989) 67(1):44–50. 2661416 PMC1385286

[89] Bosco MC Espinoza-Delgado I Schwabe M Gusella GL Longo DL Sugamura K Regulation by interleukin-2 (IL-2) and interferon gamma of IL-2 receptor gamma chain gene expression in human monocytes. Blood (1994) 83(10):2995. 8180396

[90] Hu Y Turner MJ Shields J Gale MS Hutto E Roberts BL Investigation of the mechanism of action of alemtuzumab in a human CD52 transgenic mouse model. Immunology (2009) 128(2):260–70. 10.1111/j.1365-2567.2009.03115.x 19740383 PMC2767316

[91] Zhang PL Malek SK Prichard JW Lin F Yahya TM Schwartzman MS Acute cellular rejection predominated by monocytes is a severe form of rejection in human renal recipients with or without Campath-1H (alemtuzumab) induction therapy. Am J Transplant (2005) 5(3):604–7. 10.1111/j.1600-6143.2004.00712.x 15707416

[92] Bloom D Chang Z Pauly K Kwun J Fechner J Hayes C BAFF is increased in renal transplant patients following treatment with alemtuzumab. Am J Transplant (2009) 9(8):1835–45. 10.1111/j.1600-6143.2009.02710.x 19522878 PMC4876605

[93] Lenihan CR Tan JC Kambham N. Acute transplant glomerulopathy with monocyte rich infiltrate. Transpl Immunol (2013) 29(1–4):114–7. 10.1016/j.trim.2013.09.004 24056179 PMC5091013

[94] Liu J Farmer JD Jr Lane WS Friedman J Weissman I Schreiber SL. Calcineurin is a common target of cyclophilin-cyclosporin A and FKBP-FK506 complexes. Cell (1991) 66(4):807–15. 10.1016/0092-8674(91)90124-H 1715244

[95] Fruman DA Klee CB Bierer BE Burakoff SJ. Calcineurin phosphatase activity in T lymphocytes is inhibited by FK 506 and cyclosporin A. Proc Natl Acad Sci U S A (1992) 89(9):3686–90. 10.1073/pnas.89.9.3686 1373887 PMC525555

[96] Vafadari R Hesselink DA Cadogan MM Weimar W Baan CC. Inhibitory effect of tacrolimus on p38 mitogen-activated protein kinase signaling in kidney transplant recipients measured by whole-blood phosphospecific flow cytometry. Transplantation (2012) 93(12):1245–51. 10.1097/TP.0b013e318250fc62 22643331

[97] Kang YJ Kusler B Otsuka M Hughes M Suzuki N Suzuki S Calcineurin negatively regulates TLR-mediated activation pathways. J Immunol (2007) 179(7):4598–607. 10.4049/jimmunol.179.7.4598 17878357

[98] Kono H Rock KL. How dying cells alert the immune system to danger. Nat Rev Immunol (2008) 8(4):279–89. 10.1038/nri2215 18340345 PMC2763408

[99] Rao DA Pober JS. Endothelial injury, alarmins, and allograft rejection. Crit Rev Immunol (2008) 28(3):229–48. 10.1615/CritRevImmunol.v28.i3.40 19024347

[100] Zhuang Q Lakkis FG. Dendritic cells and innate immunity in kidney transplantation. Kidney Int (2015) 87(4):712–8. 10.1038/ki.2014.430 25629552 PMC4382394

[101] Weimer R Melk A Daniel V Friemann S Padberg W Opelz G. Switch from cyclosporine A to tacrolimus in renal transplant recipients: impact on Th1, Th2, and monokine responses. Hum Immunol (2000) 61(9):884–97. 10.1016/S0198-8859(00)00152-X 11053632

[102] Zuckermann A Klepetko W Birsan T Taghavi S Artemiou O Wisser W Comparison between mycophenolate mofetil- and azathioprine-based immunosuppressions in clinical lung transplantation. J Heart Lung Transplant (1999) 18(5):432–40. 10.1016/S1053-2498(99)00004-2 10363687

[103] Rigotti P Cadrobbi R Baldan N Sarzo G Parise P Furian L Mycophenolate mofetil (MMF) versus azathioprine (AZA) in pancreas transplantation: a single-center experience. Clin Nephrol (2000) 53(4):52–4. 10809437

[104] van Gelder T Hesselink DA. Mycophenolate revisited. Transpl Int (2015) 28(5):508–15. 10.1111/tri.12554 25758949

[105] Allison AC Eugui EM Immunosuppressive and other effects of mycophenolic acid and an ester prodrug, mycophenolate mofetil. Immunol Rev (1993) 136:5–28. 10.1111/j.1600-065X.1993.tb00652.x 7907572

[106] Glomsda BA Blaheta RA Hailer NP Inhibition of monocyte//endothelial cell interactions and monocyte adhesion molecule expression by the immunosuppressant mycophenolate mofetil. Spinal Cord (2003) 41(11):610–9. 10.1038/sj.sc.3101512 14569262

[107] Jantzen HM Strahle U Gloss B Stewart F Schmid W Boshart M Cooperativity of glucocorticoid response elements located far upstream of the tyrosine aminotransferase gene. Cell (1987) 49(1):29–38. 10.1016/0092-8674(87)90752-5 2881624

[108] Rigaud G Roux J Pictet R Grange T. In vivo footprinting of rat TAT gene: dynamic interplay between the glucocorticoid receptor and a liver-specific factor. Cell (1991) 67(5):977–86. 10.1016/0092-8674(91)90370-E 1683601

[109] Cato AC Wade E. Molecular mechanisms of anti-inflammatory action of glucocorticoids. Bioessays (1996) 18(5):371–8. 10.1002/bies.950180507 8639160

[110] Newton R Holden NS. Separating transrepression and transactivation: a distressing divorce for the glucocorticoid receptor? Mol Pharmacol (2007) 72(4):799–809. 10.1124/mol.107.038794 17622575

[111] Sumegi A Antal-Szalmas P Aleksza M Kovacs I Sipka S Zeher M Glucocorticosteroid therapy decreases CD14-expression and CD14-mediated LPS-binding and activation of monocytes in patients suffering from systemic lupus erythematosus. Clin Immunol (2005) 117(3):271–9. 10.1016/j.clim.2005.09.002 16316784

[112] Orii M Imanishi T Teraguchi I Nishiguchi T Shiono Y Yamano T Circulating CD14++CD16+ monocyte subsets as a surrogate marker of the therapeutic effect of corticosteroid therapy in patients with cardiac sarcoidosis. Circ J (2015) 79(7):1585–92. 10.1253/circj.CJ-14-1422 25833081

[113] Jirapongsananuruk O Leung DY. The modulation of B7.2 and B7.1 on B cells by immunosuppressive agents. Clin Exp Immunol (1999) 118(1):1–8. 10.1046/j.1365-2249.1999.01028.x 10540152 PMC1905388

[114] Italiani P Boraschi D. From monocytes to M1/M2 macrophages: phenotypical vs. functional differentiation. Front Immunol (2014) 5:514. 10.3389/fimmu.2014.00514 25368618 PMC4201108

[115] Schif-Zuck S Gross N Assi S Rostoker R Serhan CN Ariel A. Saturated-efferocytosis generates pro-resolving CD11b low macrophages: modulation by resolvins and glucocorticoids. Eur J Immunol (2011) 41(2):366–79. 10.1002/eji.201040801 21268007 PMC3082320

[116] Poon IK Lucas CD Rossi AG Ravichandran KS. Apoptotic cell clearance: basic biology and therapeutic potential. Nat Rev Immunol (2014) 14(3):166–80. 10.1038/nri3607 24481336 PMC4040260

[117] Saas P Daguindau E Perruche S. Concise review: apoptotic cell-based therapies-rationale, preclinical results and future clinical developments. Stem Cells (2016) 34(6):1464–73. 10.1002/stem.2361 27018198

[118] Bonnefoy F Daoui A Valmary-Degano S Toussirot E Saas P Perruche S. Apoptotic cell infusion treats ongoing collagen-induced arthritis, even in the presence of methotrexate, and is synergic with anti-TNF therapy. Arthritis Res Ther (2016) 18:184. 10.1186/s13075-016-1084-0 27516061 PMC4982016

[119] Wang Z Larregina AT Shufesky WJ Perone MJ Montecalvo A Zahorchak AF Use of the inhibitory effect of apoptotic cells on dendritic cells for graft survival via T-cell deletion and regulatory T cells. Am J Transplant (2006) 6(6):1297–311. 10.1111/j.1600-6143.2006.01308.x 16686754

[120] Shipkova M Hesselink DA Holt DW Billaud EM van Gelder T Kunicki PK Therapeutic drug monitoring of everolimus: a consensus report. Ther Drug Monit (2016) 38(2):143–69. 10.1097/FTD.0000000000000260 26982492

[121] Graav GND Bergan S Baan CC Weimar W van Gelder T Hesselink DA Therapeutic drug monitoring of belatacept in kidney transplantation. Ther Drug Monit (2015) 37(5):560–7. 10.1097/FTD.0000000000000179 25551406

[122] Ford ML Adams AB Pearson TC. Targeting co-stimulatory pathways: transplantation and autoimmunity. Nat Rev Nephrol (2014) 10(1):14–24. 10.1038/nrneph.2013.183 24100403 PMC4365450

[123] Graav GND Hesselink DA Dieterich M Kraaijeveld R Douben H de Klein A An acute cellular rejection with detrimental outcome occurring under belatacept-based immunosuppressive therapy: an immunological analysis. Transplantation (2015) 37(5):560–7. 10.1097/TP.0000000000001004 26599491

[124] Dhimolea E Canakinumab. MAbs (2010) 2(1):3–13. 10.4161/mabs.2.1.10328 20065636 PMC2828573

[125] Wanderer AA Rationale and timeliness for IL-1β-targeted therapy to reduce allogeneic organ injury at procurement and to diminish risk of rejection after transplantation. Clin Transplant (2010) 24(3):307–11. 10.1111/j.1399-0012.2010.01256.x 20394637

[126] Ebert EC Infliximab and the TNF-α system. Am J Physiol Gastrointest Liver Physiol (2009) 296(3):G612–20. 10.1152/ajpgi.90576.2008 19136378

[127] Lugering A Schmidt M Lugering N Pauels HG Domschke W Kucharzik T. Infliximab induces apoptosis in monocytes from patients with chronic active Crohn’s disease by using a caspase-dependent pathway. Gastroenterology (2001) 121(5):1145–57. 10.1053/gast.2001.28702 11677207

[128] Shenghao T Yonghong H Fu’er L. Effect of sinomenine on IL-8, IL-6, IL-2 produced by peripheral blood mononuclear cells. J Tongji Med Univ (1999) 19(4):257–9. 10.1007/bf02886956 12938511

[129] Tesch GH Hill PA Wei M Nikolic-Paterson DJ Dutartre P Atkins RC. LF15-0195 prevents the induction and inhibits the progression of rat anti-GBM disease. Kidney Int (2001) 60(4):1354–65. 10.1046/j.1523-1755.2001.00940.x 11576349

[130] Lundberg S Lundahl J Gunnarsson I Jacobson SH. Atorvastatin-induced modulation of monocyte respiratory burst in vivo in patients with IgA nephropathy: a chronic inflammatory kidney disease. Clin Nephrol (2010) 73(3):221–8. 10.5414/CNP73221 20178722

[131] Mandosi E Fallarino M Gatti A Carnovale A Rossetti M Lococo E Atorvastatin downregulates monocyte CD36 expression, nuclear NFkappaB and TNFalpha levels in type 2 diabetes. J Atheroscler Thromb (2010) 17(6):539–45. 10.5551/jat.2956 20134099

[132] Davignon J-L Hayder M Baron M Boyer J-F Constantin A Apparailly F Targeting monocytes/macrophages in the treatment of rheumatoid arthritis. Rheumatology (2012) 52(4):590–8. 10.1093/rheumatology/kes304 23204551

[133] Kikuchi J Hashizume M Kaneko Y Yoshimoto K Nishina N Takeuchi T. Peripheral blood CD4(+)CD25(+)CD127(low) regulatory T cells are significantly increased by tocilizumab treatment in patients with rheumatoid arthritis: increase in regulatory T cells correlates with clinical response. Arthritis Res Ther (2015) 17:10. 10.1186/s13075-015-0526-4 25604867 PMC4332922

[134] Kaminska B MAPK signalling pathways as molecular targets for anti-inflammatory therapy – from molecular mechanisms to therapeutic benefits. Biochim Biophys Acta (2005) 1754(1–2):253–62. 10.1016/j.bbapap.2005.08.017 16198162

[135] Gosselin J Flamand L D’Addario M Hiscott J Stefanescu I Ablashi DV Modulatory effects of Epstein-Barr, herpes simplex, and human herpes-6 viral infections and coinfections on cytokine synthesis. A comparative study. J Immunol (1992) 149(1):181–7. 1318897

[136] Castro-Dopico T Clatworthy MR Fcγ receptors in solid organ transplantation. Curr Transplant Rep (2016) 3:284–93. 10.1007/s40472-016-0116-7 27909648 PMC5107199

[137] Kelly C Jefferies C Cryan S-A. Targeted liposomal drug delivery to monocytes and macrophages. J Drug Deliv (2011) 2011:11. 10.1155/2011/727241 21512579 PMC3065850

[138] Pashover-Schallinger E Aswad M Schif-Zuck S Shapiro H Singer P Ariel A. The atypical chemokine receptor D6 controls macrophage efferocytosis and cytokine secretion during the resolution of inflammation. FASEB J (2012) 26(9):3891–900. 10.1096/fj.11-194894 22651933

[139] Murray PJ Wynn TA. Protective and pathogenic functions of macrophage subsets. Nat Rev Immunol (2011) 11(11):723–37. 10.1038/nri3073 21997792 PMC3422549

[140] Zhang C Wang S Yang C Rong R. The crosstalk between myeloid derived suppressor cells and immune cells: to establish immune tolerance in transplantation. J Immunol Res (2016) 2016:4986797. 10.1155/2016/4986797 27868073 PMC5102737

[141] Brem-Exner BG Sattler C Hutchinson JA Koehl GE Kronenberg K Farkas S Macrophages driven to a novel state of activation have anti-inflammatory properties in mice. J Immunol (2008) 180(1):335–49. 10.4049/jimmunol.180.1.335 18097035

[142] Kraaij MD van der Kooij SW Reinders ME Koekkoek K Rabelink TJ van Kooten C Dexamethasone increases ROS production and T cell suppressive capacity by anti-inflammatory macrophages. Mol Immunol (2011) 49(3):549–57. 10.1016/j.molimm.2011.10.002 22047959

[143] Conde P Rodriguez M van der Touw W Jimenez A Burns M Miller J DC-SIGN(+) macrophages control the induction of transplantation tolerance. Immunity (2015) 42(6):1143–58. 10.1016/j.immuni.2015.05.009 26070485 PMC4690204

[144] Scalea JR Tomita Y Lindholm CR Burlingham W. Transplantation tolerance induction: cell therapies and their mechanisms. Front Immunol (2016) 7:87. 10.3389/fimmu.2016.00087 27014267 PMC4779899

[145] Quillard T Charreau B. Impact of notch signaling on inflammatory responses in cardiovascular disorders. Int J Mol Sci (2013) 14(4):6863–88. 10.3390/ijms14046863 23531541 PMC3645668

[146] Zhang Q Wang C Liu Z Liu X Han C Cao X Notch signal suppresses Toll-like receptor-triggered inflammatory responses in macrophages by inhibiting extracellular signal-regulated kinase 1/2-mediated nuclear factor kappaB activation. J Biol Chem (2012) 287(9):6208–17. 10.1074/jbc.M111.310375 22205705 PMC3307302

[147] Wang N Liang H Zen K. Molecular mechanisms that influence the macrophage m1-m2 polarization balance. Front Immunol (2014) 5:614. 10.3389/fimmu.2014.00614 25506346 PMC4246889

